# Reduction of High Expressed Emotion and Treatment Outcomes in Anorexia Nervosa—Caregivers’ and Adolescents’ Perspective

**DOI:** 10.3390/jcm9072021

**Published:** 2020-06-27

**Authors:** Julia Philipp, Stefanie Truttmann, Michael Zeiler, Claudia Franta, Tanja Wittek, Gabriele Schöfbeck, Michaela Mitterer, Dunja Mairhofer, Annika Zanko, Hartmut Imgart, Ellen Auer-Welsbach, Janet Treasure, Gudrun Wagner, Andreas F. K. Karwautz

**Affiliations:** 1 Eating Disorders Unit, Department of Child and Adolescent Psychiatry, Medical University of Vienna, 1090 Vienna, Austria; julia.philipp@meduniwien.ac.at (J.P.); stefanie.truttmann@meduniwien.ac.at (S.T.); michael.zeiler@meduniwien.ac.at (M.Z.); claudiaparfuss@gmx.at (C.F.); tanja.wittek@meduniwien.ac.at (T.W.); gabriele.schoefbeck@meduniwien.ac.at (G.S.); michaela.mitterer@meduniwien.ac.at (M.M.); dunja.mairhofer@meduniwien.ac.at (D.M.); gudrun.wagner@meduniwien.ac.at (G.W.); 2 Parkland Clinic, Clinic for Psychosomatic Medicine and Psychotherapy, 34537 Bad Wildungen, Germany; annika.zanko@parkland-klinik.de (A.Z.); hartmut.imgart@parkland-klinik.de (H.I.); 3 Department for Neurology and Child and Adolescent Psychiatry, 9020 Klagenfurt am Wörthersee, Austria; Ellen.Auer-Welsbach@kabeg.at; 4 Section of Eating Disorders, Department of Psychological Medicine, Institute of Psychiatry, Psychology & Neuroscience, King’s College London, London WC2R 2LS, UK; janet.treasure@kcl.ac.uk

**Keywords:** anorexia nervosa, high expressed emotion, children and adolescents, intervention, caregivers

## Abstract

High expressed emotion (EE) is common in caregivers of patients with anorexia nervosa (AN) and associated with poorer outcome for patients. In this study, we examined the prevalence of high EE in caregivers of adolescents with AN and analyzed predictors for EE using multivariate linear regression models. We further analyzed whether EE is reduced by the “Supporting Carers of Children and Adolescents with Eating Disorders in Austria” (SUCCEAT) intervention using general linear mixed models and whether a reduction of EE predicts patients’ outcomes. Caregivers were randomly allocated to the SUCCEAT workshop (*N* = 50) or online intervention (*N* = 50) and compared to a comparison group (*N* = 49). EE and patients’ outcomes were assessed at the baseline, post-intervention, and at the 12-month follow-up. Up to 47% of caregivers showed high EE. Lower caregiver skills, higher AN symptom impact, higher levels of depression and motivation to change in caregivers were significant predictors for high EE. EE significantly decreased in the SUCCEAT groups and the comparison group according to the caregivers’, but not the patients’ perspective. The level of reduction could partially predict subjective improvement and improvement in clinically assessed AN symptoms and body mass index of patients. Implementing interventions for caregivers addressing EE in the treatment of adolescents with AN is strongly recommended.

## 1. Introduction

Expressed emotion (EE) is a concept that describes a set of attitudes and behaviors (e.g., criticism, hostility, warmth, emotional involvement) of relatives towards an ill person. Specifically, the components of EE that have been mainly investigated so far are critical comments/criticism (CC) and emotional overinvolvement (EOI) [1,2]. High EE may represent a maladaptive response to an illness and may contribute to an exacerbation of psychiatric symptoms [2]. EE has been extensively studied in schizophrenia and has been confirmed to be a robust and significant predictor of relapse [3]. High levels of EE have further consistently been associated with treatment outcome, relapse, and treatment dropout in several other psychiatric disorders, including depression, bipolar disorder, anxiety disorders, and also eating disorders (EDs) [1,2,3].

In the field of EDs, caring for someone with an ED is known to be associated with high EE [1,4,5]. Caregivers often show extreme patterns of emotional reactions [6]. They might respond too intensely and too emotional, be too directive, negative and hostile, and blame the patient (CC), or they can be overprotective, overinvolved, and take over control (EOI). In a systematic review, up to 73.2% of caregivers showed high levels of CC and 89.3% showed high levels of EOI [5]. High EE has been found to be associated with caring for older patients and longer illness duration [4], higher anxiety and depression in caregivers [7], more contact time with the patient [8], higher caregivers’ distress [8,9], and less caregiver skills [9]. Other studies revealed no clear association of high EE with the patients’ weight or ED psychopathology [1,10], time spent caregiving, and ED severity [9]. Only few studies investigated the ED patients’ point of view of EE within the family. Perceived EE in the family assessed by ED patients was reported to be higher than in healthy controls [11]. Patients reported higher EOI for their mothers, but no difference between mothers and fathers for CC [9]. The level of perceived EE from the patients’ point of view was independent from age, contact time, and duration of treatment [11]. However, it has consistently been shown that high levels of EE in caregivers, especially in mothers, seem to negatively affect engagement, outcome, and effectiveness of ED treatment [1,2,3,10,12,13]. Thus, considering EE of caregivers in the patients’ treatment seems to be highly relevant.

The key role of interpersonal relationships, particularly, communication and high EE within the family has also been highlighted in the interpersonal maintenance model of anorexia nervosa (AN) [14,15,16]. EE was related to caregivers’ distress. Caregivers’ distress was related to patients’ distress, which predicted eating disorder symptoms [8]. Interventions based on this model were developed to reduce high EE [17] in order to improve the psychological wellbeing in caregivers and support recovery from the ED in patients [4]. These interventions address misperceptions of EDs and unhelpful reactions to the illness [18] and aim to improve communication skills, reduce negative emotion and build a warm and compassionate family atmosphere [19]. Caregivers learn to use reflective listening and motivational interviewing techniques, reduce confrontation, and be warm, calm, and compassionate.

A meta-analysis showed a moderate-sized reduction of EE in caregivers after participating in such interventions, including workshop-based, online, and self-help interventions [18]. Other recent studies also reported a reduction of high EE following interventions for caregivers [20,21,22]. However, having investigated the effects of such interventions, the previous literature almost exclusively focused on the parents’ perception, while not considering the perception of the patients, although including the patients’ perception is recommended and valued to gain additional insight in the family climate of ED patients [1]. One study that included the patients’ perspective found that patients did not perceive a change in their caregivers’ level of EE after caregivers participated in a specialized intervention [23], while in another study, qualitative improvement in caregivers’ EE was mentioned by patients [24].

So far, only few studies have investigated whether a reduction in EE can also improve the patients’ treatment outcomes. Two studies reported that an improvement of maternal EE was associated with better ED symptoms at the end of the treatment [10,25]. However, whether the level of reduction in parental EE is associated with the level of reduction in ED symptoms has not been investigated up to now.

SUCCEAT (Supporting Carers of Children and Adolescents with Eating Disorders in Austria) is an intervention for caregivers of adolescents with AN based on the interpersonal maintenance model [14,15,16] that has already been shown to effectively reduce caregiving burden and increase caregiver skills [26]. This paper presents secondary analyses of the SUCCEAT study, focusing on various aspects of high EE. Firstly, we aimed to examine the prevalence of high EE in a sample of caregivers and whether the level of EE assessed in parents is associated with the level of EE assessed in patients. Secondly, we explored whether different characteristics of caregivers and patients can predict the level of EE. Thirdly, this study aims to investigate whether EE can be reduced by participating in the SUCCEAT intervention and whether a change in assessed parental EE is also perceived by patients. Finally, we aimed to investigate whether a reduction in high EE is associated with patients’ outcomes (AN symptoms, body mass index (BMI)).

## 2. Methods

### 2.1. Study Design

The SUCCEAT study is a two-arm parallel-group quasi-randomized controlled efficacy trial comparing two types of the SUCCEAT intervention (workshop and online) to a non-randomized control group. This study was conducted between 2014 and 2019. Caregivers of children and adolescents (10–19 years) with typical or atypical AN who received regular inpatient or outpatient treatment according to the National Institute for Health and Care Excellence (NICE) guidelines [27] were included. Caregivers and patients who were not fluent in German or suffered from severe comorbidities (e.g., psychosis) were excluded. For detailed information on the study protocol, see Franta et al. [28]. Written informed consent was obtained from all the participants prior to data collection. The SUCCEAT study protocol and all the informed consent forms were approved by the Ethics Committee of the Medical University of Vienna (#1840/2013). The SUCCEAT study was registered at ClinicalTrials.gov (Identifier: NCT02480907).

### 2.2. Recruitment and Randomisation

Caregivers of the SUCCEAT intervention group were recruited at the Eating Disorder Unit of the Department of Child and Adolescent Psychiatry at the Medical University of Vienna. If willing to participate, caregivers completed the baseline assessment and were allocated to one of the two intervention arms: workshop (SUCCEAT–WS) or online (SUCCEAT–ONL). The start dates of the groups had been previously fixed. The first block of participants was allocated to the SUCCEAT–WS group, the following block—to the SUCCEAT–ONL group, and so forth. Block sizes of eight participants were planned, however, sizes actually varied due to varying numbers of incoming participants prior to the upcoming group (median group size = 7). Caregivers were informed about the group allocation after they had completed the baseline assessments. Those who declined to participate in the allocated intervention arm were excluded from the study. The procedure slightly differed from the originally planned randomization [28] due to practical reasons, now corresponding to the definition of quasi-randomization [29]. However, there were no systematic differences between the WS and ONL groups at the baseline. Details on the randomization procedure are reported elsewhere [26].

### 2.3. Interventions

SUCCEAT is an intervention for caregivers of children and adolescents with AN based on the cognitive interpersonal model of maintaining factors for EDs [14,15,16] with the aim to reduce EE and burden in caregivers and improve caregiver skills. Dysfunctional communication, unhelpful reactions and emotions were addressed to increase communication skills, skills to handle difficult behavior, and strategies to encourage autonomy in patients in order to reduce EE. Additionally, caregivers’ burden, wellbeing, and self-care were addressed as burden and psychopathology had been highly correlated with EE. The program was delivered in eight weekly modules as a WS or ONL, designed with the same content and structure and guided by two professional clinicians. Caregivers received weekly handouts, a manual to read more about the topics addressed [30], and a DVD [31] with case examples of unhelpful and helpful communication. A detailed description of the interventions is provided by Franta et al. [28] and Truttmann, Philipp et al. [26].

### 2.4. Comparison Group

The SUCCEAT groups were compared to a non-randomized comparison group (COMP) of caregivers who received other forms of family treatment within the same time frame at two other facilities: the Department for Neurology and Child and Adolescent Psychiatry, Klagenfurt, Austria (four double sessions of systemic family therapy [32]), and the Parkland Clinic, Clinic for Psychosomatic Medicine and Psychotherapy, Bad Wildungen, Germany (two-day workshop on multi-family therapy based on the Maudsley model of multi-family therapy for AN [33]). Several other inpatient and outpatient units that offer treatment as usual with and without any specialized intervention for caregivers were invited to participate as the control group declined the offer. Therefore, only the two named facilities that already implemented well-established family treatment agreed to participate and served as the COMP group.

### 2.5. Assessment Measures

Caregivers and patients completed self-report questionnaires at baseline (T0), at post-intervention (T1, approximately 3 months after T0), and at 12-month follow-up (FU) (T2), including the assessment of EE described below.

The Family Questionnaire (FQ [34]) is a self-report questionnaire for caregivers to detect the emotional climate and EE within the family. The 20 questions were answered using a four-point Likert scale providing two subscales of EE in caregivers: “Emotional overinvolvement” (EOI) and “Criticism” (CC). We used the established cut-off values (CC score ≥ 23, EOI score ≥ 27) to define high EE.

The Family Emotional Involvement and Criticism Scale (FEICS [35]) is a self-report questionnaire for children and adolescents to assess perceived EE within the family. Two subscales were calculated (“Emotional Involvement” and “Criticism”) using 14 questions that were answered using a four-point Likert scale. For the FEICS scores, no established cut-off values are available.

In addition, self-assessments for caregivers were used to explore predictors of high EE and associations with perceived changes as described below (for details, see Franta et al. [28]).

The General Health Questionnaire (GHQ [36]) was fulfilled by caregivers and used to assess the level of psychological morbidity and distress in caregivers; a higher score indicates higher psychological distress.

The Eating Disorder Symptom Impact Scale (EDSIS [37]) assessed specific caregiving difficulties in caregivers associated with the ED of a child, such as difficulties related to “nutrition”, “dysregulated behavior”, “guilt”, and “social isolation.” A higher global score represents higher AN symptom impact in the family reported by caregivers.

The Beck Depression Inventory (BDI-II [38]) measured depression in caregivers; higher scores indicate higher levels of depression.

The State and Trait Anxiety Inventory (STAI [39]) assessed anxiety in caregivers; higher scores indicate higher levels of anxiety.

The Caregiver Skills (CASK) Scale [17] was used to measure self-assessed skills in caregivers (e.g., “insight and acceptance”, “emotional intelligence”, “frustration tolerance”). A higher total score means more caregiver skills.

The University of Rhode Island Change Assessment Scale (URICA [40]) was used to measure motivation to change in caregivers regarding their own behavior, revealing three scales: “precontemplation”, “contemplation”, and “action.” A lower score on the precontemplation scale and higher scores on the contemplation and action scales represent higher motivation to change in caregivers.

Finally, two more assessments for patients were used as described below.

The Eating Disorder Examination (EDE [41]) is a semi-structured interview with patients reflecting the clinical assessment of ED psychopathology (“restraint”, “eating concerns”, “weight concerns”, “shape concerns”). A higher global score indicates higher ED psychopathology.

The Eating Disorder Inventory-2 (EDI-2 [42]) was completed by the patients and assessed subjective ED symptoms from the patients’ perspective (e.g., “drive for thinness”, “perfection”, “dissatisfaction with the body”, etc.). A higher total score indicates more subjective ED symptoms.

Sociodemographic and clinical characteristics of caregivers (sex, age, marital status, highest educational degree, time spent caregiving) and patients (sex, age, AN subtype, BMI percentile, illness duration, type of current treatment) were obtained.

### 2.6. Statistical Analysis

The statistical analyses were performed using IBM SPSS Statistics 25.0 and R. We first calculated descriptive statistics and compared key sociodemographic and clinical characteristics between caregivers and patients of the SUCCEAT–WS, SUCCEAT–ONL, and the COMP group. We used Chi² tests for categorical and ANOVA tests for continuous variables, respectively, and Kruskal–Wallis tests for variables with skewed distribution. We calculated *t*-tests to analyze differences in CC and EOI scores between a mother and a father and Pearson correlation coefficients to explore associations between CC and EOI obtained from the parents and patients. To analyze predictors for assessed parental and patients’ CC and EOI scores at the baseline, we first carried out a series of univariate linear regressions. The following predictors were considered: sex and age of the parent, parental psychological distress and psychopathology (including the GHQ, BDI, and STAI scores), AN symptom impact on the family (EDSIS score), self-reported caregiver skills (CASK score), parental motivation to change (URICA scores), the average time spent caregiving per day (<3 h vs. ≥3 h), the patient’s age, treatment type (inpatient vs. outpatient), ED duration in months, BMI percentile (≤1st percentile vs. >1st percentile) and ED symptomatology (EDE and EDI-2 total scores). Variables reaching significance in univariate regressions (*p* < 0.05) were then considered for multivariate regression models using the forward selection method where variables were added to the model of ascending *p*-values.

In order to analyze the efficacy of the SUCCEAT intervention on EE, we calculated general linear mixed models using the FQ/FEICS scores (CC and EOI) obtained at the baseline, post-intervention, and at 12-month FU as the within factor and the group as the between factor. Firstly, we contrasted the SUCCEAT–WS group to the SUCCEAT–ONL intervention group. Secondly, we tested whether the SUCCEAT intervention (including all WS and ONL participants) differ from participants of the COMP group. Additionally, we performed sensitivity analyses by calculating paired sample *t*-tests for each group to explore the baseline-to-post-intervention and the baseline-to-12-month FU effect sizes (Cohen’s dz). Furthermore, we explored the moderating effect of the patient’s treatment type (inpatient vs. outpatient) on the EE outcome by adding a time x treatment type interaction term to the mixed design model.

Further, we explored whether a pre–post change in assessed parental CC and EOI scores corresponds to a change in CC and EOI assessed in the patients by calculating Pearson correlation coefficients.

Finally, we used linear regression analyses to analyze whether the degree of reduction in CC and EOI can predict improvements in ED symptomatology of the patients by using the EDE and EDI-2 change scores as well as the change in the BMI percentile as outcome variables and CC/EOI change scores as predictor variables.

## 3. Results

### 3.1. Sample

In total, 149 caregivers (83% mothers, 17% fathers; mean age (SD): 47.2 (4.74) years) provided informed consent and were included in the study. Forty-five percent had a university degree and 77% were married or lived in a partnership. According to their self-reports, the average amount of time spent with their child diagnosed with AN during weekdays was distributed as follows: 0–1 h (10%), 1–3 h (19%), 3–4 h (35%), > 4 h (35%). A total of 50 caregivers were allocated to the SUCCEAT–WS intervention, another 50 caregivers—to the SUCCEAT–ONL intervention, and 49 caregivers were included in the COMP group.

Of the 149 caregivers participating, we obtained data from 144 related patients with AN. The patients were predominantly female (83%) with a mean age of 15.1 years (SD: 1.7). According to ICD-10, most patients were diagnosed with AN restrictive subtype (89%), followed by AN binge/purging subtype (9%) and atypical AN (2%). At the baseline, the median sex-and age-specific BMI percentile was 1%, the average ED duration was 16.6 months (SD: 13.8), 59% received inpatient treatment, and 41% received outpatient treatment of their ED.

Baseline sample characteristics by intervention arm are shown in Table 1. There was a significantly lower proportion of caregivers with university degree in the COMP and patients of caregivers in the COMP had a significantly longer ED duration and higher subjective ED symptomatology scores as measured with EDI-2 and received inpatient treatment more often compared to SUCCEAT groups.

Dropout of caregivers in terms of non-completion at one of the FU assessments was 14.8% at the post-intervention assessment (SUCCEAT–WS: 4.0%, SUCCEAT–ONL: 6.0%, COMP: 34.7%) and 29.6% at the 12-month FU assessment (SUCCEAT–WS: 14.0%, SUCCEAT–ONL: 28.0%, COMP: 46.9%). Dropout of patients was 13.9% at the post-intervention assessment (SUCCEAT–WS: 8.3%, SUCCEAT–ONL: 6.1%, COMP: 27.7%) and 35.4% at the 12-month FU assessment (SUCCEAT–WS: 31.2%, SUCCEAT–ONL: 32.7%, COMP: 42.6%). Caregivers and patients who dropped out did not significantly differ from those who completed the study regarding the baseline EE scores (all *p*-values > 0.200).

### 3.2. Baseline EE Characteristics

Among the total sample, high CC defined as scores above the pre-defined cut-off in the FQ were observed in 39.2% (95% CI: 31.3; 47.1) and high EOI—in 46.6% (95% CI: 38.5; 54.7) of caregivers. Mean baseline CC and EOI scores obtained from the parents and patients are shown in Table 1. Mothers had slightly higher CC (mean: 21.79, SD: 5.52) and EOI scores (mean: 26.56, SD: 5.18) compared to fathers (CC: mean: 18.87, SD: 5.48; EOI: mean: 24.74, SD: 4.43); however, these differences did not reach statistical significance (CC: *t* = 1.613, *p* = 0.109; EOI: *t* = 1.672, *p* = 0.097).

### 3.3. Bivariate Correlations between Parents’ and Patients’ EE Scores

At the baseline, the parental EE scores were not significantly associated with the EE scores obtained from the patients’ perspective neither for CC (*r* = 0.078, *p* = 0.356) nor for EOI (*r* = 0.115, *p* = 0.171). This correlation slightly increased at post-intervention (CC: *r* = 0.142, *p* = 0.126; EOI: *r* = 0.117, *p* = 0.206) and 12-month FU (CC: *r* = 0.216, *p* = 0.042; EOI: *r* = 0.269, *p* = 0.011). Considering mothers and fathers separately, we found a strong association between CC scores of fathers and patients at the baseline (*r* = 0.509, *p* = 0.009), while there was no association between CC scores of mothers and patients (*r* = 0.006, *p* = 0.946). No difference between mothers and fathers were found for EOI scores.

### 3.4. Predictors for EE Scores at the Baseline

Firstly, we explored whether different characteristics of parents and patients are predictive of the EE scores obtained from the parents. In the univariate regression analyses (Table S1 in the electronic Supplementary Materials), we found that the level of parental psychological distress, AN symptom impact, depression, anxiety, caregiver skills, parental motivation to change, and the current treatment setting (inpatient vs. outpatient) of the child were significantly associated with either the CC or EOI score or both. In the multivariate regression model (Table 2), lower caregiver skills, higher level of AN symptom impact, and an outpatient treatment setting were significant predictors for CC with the final model explaining 46.2% of the variance. The parental EOI score was significantly predicted by higher levels of AN symptom impact, higher distress, depression, as well as higher motivation to change and lower caregiving skills with the final model explaining 63.5% of the variance.

We repeated this analysis using the EE scores obtained from the patients’ perspective as the outcome variable. In the univariate regression analyses (Table S2 in the electronic Supplementary Materials), ED duration and ED symptomatology as measured with EDE and EDI-2 were significantly associated with CC and parental motivation to change and BMI were significantly associated with EOI. In the multivariate regression model (Table 3), only higher ED symptomatology (EDI-2 score) significantly predicted CC explaining 14.2% of the variance. The EOI score was significantly predicted by lower BMI (<1st BMI percentile) and lower parental motivation to change. However, the final model only explained 8.1% of the variance.

### 3.5. Intervention Outcomes on EE

**Figure 1:**
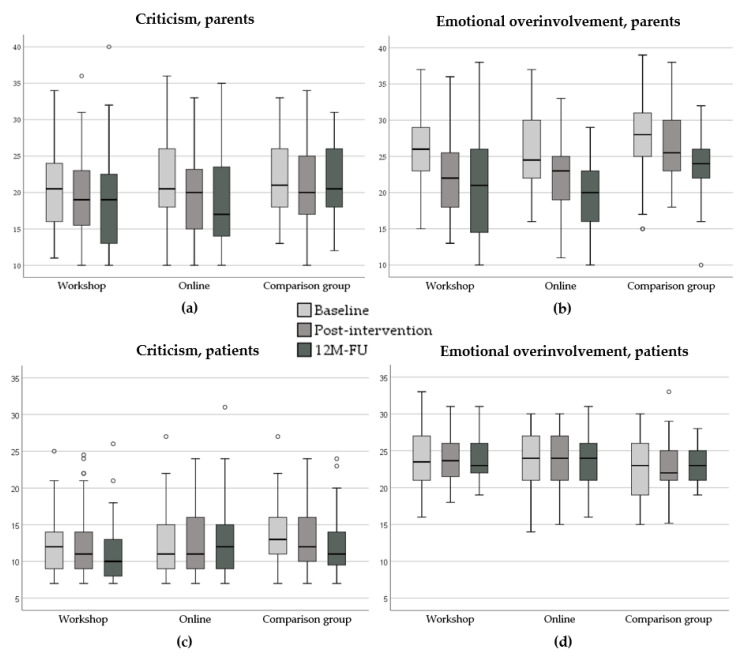
Change of high expressed emotion scores in the SUCCEAT workshop, online, and comparison groups from the baseline (light-grey box) to the post-intervention (mid-grey box) and the 12-month follow-up (dark-grey box) assessment: (**a**) Criticism score (parent perspective), (**b**) Emotional Overinvolvement score (parent perspective), (**c**) Criticism score (patient perspective), (**d**) Emotional Overinvolvement score (patient perspective). The size of the box represents the interquartile range (IQR); the whiskers indicate the minimum/maximum values in case no outliers were observed. Outliers (defined as values > 1.5 x IQR from the 25th quantile and 75th quantile) are depicted as circles. Abbreviations: 12M-FU, 12-month follow-up.

The change in EE scores by group from the baseline to the post-intervention and the 12-month FU assessment is shown in Figure 1. We first tested whether the SUCCEAT–WS group differs from SUCCEAT–ONL group. We found that the assessed parental CC (F = 7.391, *p* = 0.001) and EOI scores (F = 45.704, *p* < 0.001) significantly decreased over time across both groups, but there was no significant time × group interaction effect (CC: F = 0.437, *p* = 0.782; EOI: F = 0.953, *p* = 0.435). Considering the patients’ perspective, there was no significant main effect of time and group difference neither for CC nor for EOI.

When contrasting the SUCCEAT groups to the COMP group, we found the same pattern of results. The assessed parental CC (F = 5.400, *p* = 0.005) and EOI scores (F = 36.112, *p* < 0.001) significantly decreased over time across the groups, but this change was the same for the SUCCEAT and COMP groups (CC: F = 0.089, *p* = 0.914; EOI: F = 1.612, *p* = 0.202). Again, no significant effects of time and time x group interaction effects were observed when considering the patients’ perspective.

Sensitivity analyses using *t*-tests revealed that for the assessed parental CC, baseline-to-post-intervention reductions were highest for the SUCCEAT–ONL group (dz = 0.44, *t* = 3.788, *p* < 0.001) compared to the SUCCEAT–WS (dz = 0.20, *t* = 1.806, *p* = 0.077) and COMP groups (dz = 0.32, *t* = 2.172, *p* = 0.038). These effects remained stable to the 12-month FU for the SUCCEAT groups (WS: dz = 0.24; ONL: dz = 0.54) and slightly decreased in the COMP group (dz = 0.20). For the assessed parental EOI, baseline-to-post reductions were slightly higher for the SUCCEAT groups (WS: dz = 0.68, *t* = 5.739, *p* < 0.001; ONL: dz: 0.76, *t* = 5.927, *p* < 0.001) compared to the COMP group (dz = 0.36; *t* = 2.061, *p* = 0.048). The effect sizes further increased at the 12-month FU in all the groups (WS: dz = 0.76, ONL: dz = 1.11, COMP: dz = 0.89). Considering the patients’ perspective, all effect sizes were close to zero in all the groups.

Further analyses revealed that the type of patients’ treatment (inpatient vs. outpatient) was a significant moderator of the intervention outcome. Caregivers of outpatients reported a significantly higher reduction in CC scores than caregivers of inpatients (time x treatment type interaction: F = 4.076, *p* = 0.018). Regarding EOI, reductions of EOI scores were reported to be faster (from the baseline to post-intervention) in caregivers of outpatients, whereas the reduction was delayed (post-intervention to the 12-month FU) in caregivers of inpatients (time x treatment type interaction: F = 5.011, *p* = 0.008). This moderator effect was independent of the group.

**Figure 2:**
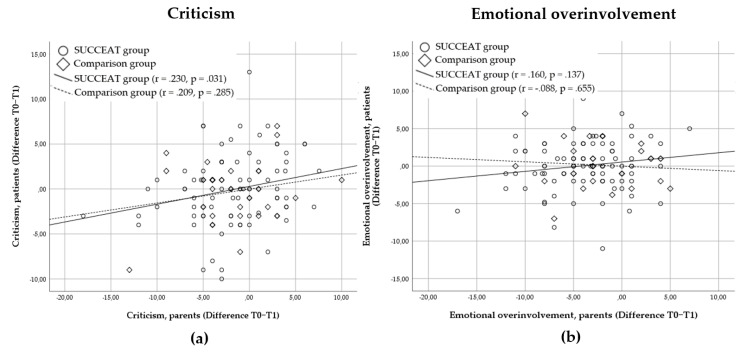
The scatterplot depicting the association (Pearson correlation coefficients) between high expressed emotion change scores (baseline to post-intervention) of parents and patients: (**a**) Criticism change scores; (**b**) Emotional overinvolvement change scores. A significant positive correlation indicates that higher levels of change based on the parents’ perspective are associated with higher levels of change based on the perspective of patients.

Figure 2 presents the bivariate correlations between the baseline-to-post-intervention change of parental and patients’ CC and EOI scores. In the SUCCEAT group, parental change of CC was moderately associated with the change of CC in patients (*r* = 0.230, *p* = 0.031). In the COMP group, this association was not significant (*r* = 0.209, *p* = 0.285). For EOI, no significant associations in the change scores were observed (SUCCEAT: *r* = 0.160, *p* = 0.137; COMP: *r* = −0.088, *p* = 0.655).

### 3.6. Impact of EE Change on the Patient’s ED Symptomatology

Table 4 shows whether a reduction in parental or patients’ EE scores significantly predicted improvements in ED symptoms (EDE and EDI-2 scores, BMI percentile) for the total sample and separately for inpatients and outpatients. We found that for the total sample, improvements in CC were significantly associated with improvements in ED symptomatology and BMI. On the contrary, reductions in EOI did not significantly predict improvements in ED symptoms.

## 4. Discussion

This study explored EE in caregivers and adolescents with AN at the baseline and following a skills training for caregivers, including a long-term FU. In addition, we aimed to investigate whether changes in EE affect patients’ outcomes.

Firstly, the caregivers’ perspective of EE was analyzed. One third of caregivers showed elevated CC scores and nearly half of the caregivers reported high EOI scores. These prevalence figures are somewhat lower compared to previous studies using the same questionnaire [5,7]. This may be due to older patients (mostly adults) and a longer illness duration in these reports (mostly far longer than one year) compared to our participants (approximately one year). Even though we only observed a tendency that longer illness duration is associated with higher EE scores in this study, this association can be assumed from the literature [4,5,10]. CC and EOI scores tended to be higher for mothers than for fathers, albeit these results did not reach statistical significance due to the small number of fathers included in this study. This is not fully in line with several previous reports describing mothers to be more overinvolved than fathers [5,9,43] and fathers to be more critical than mothers [5,43].

Low perceived caregiver skills, high AN symptom impact on caregivers, and an outpatient treatment setting were significant factors associated with CC, accounting for nearly 50% of the variance. High AN symptom impact on caregivers, depression in caregivers, higher psychological distress, higher motivation to change in caregivers, and less caregiver skills accounted for over 60% of the variance of EOI. Other studies also found associations of high EE with psychological distress or depression [1,43,44]. Caregivers’ distress and self-care should therefore be the core elements in interventions for caregivers, beside emotion regulation strategies and communication skills. Less perceived skills were associated with both higher CC and higher EOI. This may reflect that caregivers who feel less competent to deal with ED in the family demonstrate high EE, probably because they feel overwhelmed with this situation. Consequently, one might also assume that enhancing caregivers’ skills can have a positive impact on EE. Interestingly, higher motivation to change in parents was associated with higher EOI. This sounds paradoxical. However, caregivers who experience frustration and helplessness when confronted with ED symptoms or associated conflicts more often, which may consequently enhance EE, might also be highly motivated to change their own behavior or aspects of the actual situation. Therefore, it may be of benefit to target motivation to change in interventions for caregivers. Further research is needed to investigate the role of motivation to change in caregivers regarding high EE. Caregivers of outpatients reported higher scores for CC than caregivers of inpatients. It might be assumed that parents of outpatients feel more responsible for an improvement of symptoms compared to inpatients, whereas the responsibility for symptom improvement is delegated to the ED treatment team. This may lead to higher distress and burden in parents of outpatients which may promote more critical comments. Besides, an outpatient treatment setting might induce more situations where high EE can occur compared to an inpatient setting. In contrast to the literature [43], there was no association with illness severity (patients with lower BMI or higher ED psychopathology). No associations were also found for age of caregivers and age of patients, with only a tendency for caregivers of older patients to report higher EOI scores, in contrast to other studies reporting significantly higher EE in caregivers of older patients [4,5]. This may be because the mean age of patients within those studies was higher than in our sample. As the patient’s age and duration of illness often correlate, we assume that including adult patients in the study may lead to a similar result. The time spent caregiving did not play a role in our study regarding EE levels either, in contrast to another study [8]. This study included adult patients and concluded that caring for patients that live in the same household involves more time and leads to higher EE. However, as we only included adolescents, the great majority of whom still lived at home, the time spent caregiving may not play such an important role.

We also explored predictors of high EE based on the patients’ perspective. Regarding the patients’ point of view on EE, high ED psychopathology was solely associated with CC, whereas a lower BMI and higher motivation to change in caregivers were associated with EOI. Therefore, a somewhat more severe illness (lower BMI and higher ED psychopathology) was correlated with high EE in the family. Moreover, when parents were more motivated to change, the perceived EOI was higher, which is similar to the findings for parents described above. However, our model only accounted for 4% (CC) and 8% (EOI) of the variance. Hence, other factors associated with the patients’ perception of EE in the family should be investigated in future research.

Another aim of the study was to explore whether parental and patients’ perceptions of EE are correlated. Regarding the total group, there were no significant correlations at the baseline, while the correlations between caregivers and patients slightly increased with time and reached significance at the 12-month FU. It is unclear why parents and patients’ perceptions are stronger associated with time, even though the patients’ perception of high EE within the family did not decrease like the caregivers’ perception did. We assume that either the patients’ or the parents’ ability to precisely assess the real family climate improves with time, probably due to successful weight restitution or less severe symptoms at T2 or because patients gain valuable insights on family dynamics during their ongoing psychotherapy. However, the low correlations indicate that the parent’s and patient’s perceptions of EE diverge, which can be targeted in family-based interventions. Interestingly, we found a significant high correlation between the fathers’ and patients’ perceptions of CC at the baseline and at post-intervention which has to be interpreted with caution due to the low number of fathers included in this study.

We further investigated the efficacy of the SUCCEAT intervention on EE in caregivers. A significant reduction of EE was observed in the SUCCEAT group for the short-term and the long-term FU. This is in line with the literature, showing that EE in caregivers can be reduced after participating in an intervention for caregivers including psychoeducation and skills training [18,20,21,45]. EE was also reduced in the COMP group with no significant difference to the SUCCEAT intervention. That might be because the facilities that recruited participants for the COMP group already implemented specialized family interventions (multi-family therapy [33] or systemic family therapy [32]) also including expressing emotions, conflict management, and communication within the family as the core elements. To note, there were no differences between the three study arms with regard to the majority of caregivers’ and patients’ sociodemographic and clinical characteristics at the baseline. However, the groups differed with respect to the highest educational degree, ED duration, treatment setting, and subjective ED symptoms with the caregivers of the SUCCEAT group having higher educational levels and the COMP patients having a longer illness duration, higher subjective ED psychopathology, and having been treated in an inpatient setting more often. This should be considered when interpreting the results regarding group differences. Yet, there was no difference for EE scores at the baseline between the three arms. The sensitivity analyses showed that reductions in EOI were generally higher than in CC and that intervention effects remained stable or increased further up to the 12-month FU except for CC in the COMP group where we found a small rebound effect indicating that parents of the COMP group were a bit more critical at the 12-month FU again. Indeed, the current literature suggests that CC is more susceptible to increase again in the long term [43]. Interestingly, the improvement of CC and EOI was stronger in caregivers of outpatients than of inpatients and EOI also improved faster. This might be due to the fact that caregivers of outpatients may have more time and more opportunities to use and practice the communication skills trained in the interventions than caregivers of inpatients.

Unfortunately, no improvements were found when considering the patients’ perception of EE within the family. Thus, the patients did not perceive EE to be significantly reduced over time neither in the SUCCEAT nor in the COMP groups. This could be due to several reasons. Firstly, we used questionnaires that measure the same construct, but did not include exactly the same item contents, impeding direct comparisons. The FQ measures subjective CC and EOI towards the patient, whereas the FEICS assesses CC and EOI within the whole family from the patient’s point of view, but not specifically towards the parent who completed the FQ. Secondly, in most cases, only one parent (either mother or father) participated in the intervention. It might be the case that the caregivers who did not participate did not change regarding their EE compared to the ones that participated and therefore they may still contribute to a highly negative emotional climate. Thus, if possible, both parents should be encouraged to participate in a caregiver’s skills training and be included in further research. Another possible explanation is that parents feel a reduction of EE more intensely, whereas patients do not experience small changes. Rienecke [1] assumed patients with AN to be acutely sensitive to parental EE, so they may have difficulties to experience a change even if the frequency of EE has reduced. In that case, children and adolescents might need more information on EE themselves in order to be more sensitive to notice a change in the family climate [46]. It might be possible to encourage patients themselves to read some chapters of the manual or to watch some parts of the DVD used in the intervention. Another possibility is to arrange one family meeting where this topic can be discussed together or to implement this issue in the patients’ therapy.

Although there was no reduction in EE from the patients’ point of view, we found a significant association between the perceived change in CC in parents and the perceived change in CC in patients from the baseline to post-intervention in the SUCCEAT group only. However, this correlation was marginal. No such association was observed for EOI. These results were similar to another study [23] that reported a significant reduction of EE in caregivers, no perceived reduction in patients, but a positive correlation between changes in perceived EE between caregivers and patients. Furthermore, patients might experience qualitative improvements in EE as indicated by Goddard et al. [24] which might not have been covered with the questionnaire used in this study.

Finally, we investigated whether reductions in parental or perceived EE affect the outcome of ED pathology and BMI in AN patients. Indeed, improvement in the assessed parental CC was associated with greater changes in subjective ED symptoms. Similarly, greater improvement in CC assessed in patients was associated with greater changes in subjective ED symptoms, clinically assessed ED psychopathology, and BMI. For EOI, no clear association was observed. These results contribute to the literature indicating that a reduction in parental EE is associated with patients’ outcomes [10,25]. Although our analyses were explorative, these results indicate that reducing EE through a caregiver skills training can positively affect the ED psychopathology and BMI in patients. These results highlight the benefit and need in a caregiver training in the routine ED treatment.

This study has a couple of strengths. It is the first study investigating whether a change in EE can predict a change in ED psychopathology and BMI in adolescent patients. A long-term outcome was included. Both SUCCEAT groups were uniformly designed and guided by the same two professional clinicians. The study has some limitations, too. The number of fathers included in this study was low. Therefore, the role of fathers and the correlation between patients and both parents as well as between mothers and fathers require further investigation. Furthermore, as ED duration and treatment setting (outpatient or inpatient setting) differ significantly between the three groups at the baseline, results regarding group differences should be interpreted with some caution.

## 5. Conclusions

Our study confirms that high EE is common in caregivers of adolescent patients with AN. It further highlights that EE can be reduced after interventions for caregivers and that this reduction positively influences patients’ outcomes. Our results therefore underline the importance of including a focus on parental EE into the treatment of adolescents with AN. Parental EE was high and was associated with higher distress, higher depression, and a lack of skills. We found that parental EE was significantly reduced after participating in the SUCCEAT intervention or a specialized family intervention and that this reduction remained in the long term. Although the patients’ perceptions did not match well with the caregivers’ perceptions of EE, there was some evidence that reductions in EE positively affect the patient’s treatment course. Altogether, we can strongly recommend to include interventions for caregivers, such as SUCCEAT, into the treatment of adolescents with AN in order to improve the outcome for caregivers and for patients. Patients might further benefit from a conjoint meeting with parents to discuss family climate and to get sensitive to perceive possible changes.

## Figures and Tables

**Table 1:** Sample characteristics at the baseline (caregivers and eating disorder patients).

	SUCCEAT–WS (*N* = 50)	SUCCEAT–ONL (*N* = 50)	COMP (*N* = 49)	*p*
**Caregivers’ characteristics**				
Mothers (%)	84.0%	88.0%	75.5%	0.248 ^1^
Age (Mean, SD)	46.64 (5.43)	47.72 (4.25)	47.27 (4.48)	0.523 ^2^
University degree (%)	60.0%	46.0%	27.7%	0.006 ^1^
Married or living in partnership (%)	84.0%	75.0%	70.2%	0.264 ^1^
**Time spent with the patient during weekdays (%)**				
0–1 h/day	6.1%	10.2%	15.2%	0.386 ^1^
1–2 h/day	18.4%	22.4%	15.2%	
3–4 h/day	32.7%	42.9%	30.4%	
>4 h/day	42.9%	24.5%	39.1%	
FQ–CC score (Mean, SD)	20.76 (5.96)	21.78 (5.47)	21.82 (5.54)	0.559 ^2^
FQ–EOI score (Mean, SD)	26.10 (5.17)	25.44 (4.98)	27.22 (5.05)	0.222 ^2^
**Patients’ characteristics**				
Females (%)	90.0%	96.0%	100%	0.061 ^1^
Age (Mean, SD)	14.66 (1.91)	15.12 (1.80)	15.43 (1.08)	0.068 ^2^
**ED diagnosis (%)**				
AN restrictive	90.0%	90.0%	87.8%	0.995 ^1^
AN binge/purging	8.0%	8.0%	10.2%	
Atypical AN	2.0%	2.0%	2.0%	
ED duration (Mean, SD)	10.41 (7.10)	16.03 (16.05)	23.77 (12.93)	<0.001 ^1^
BMI percentile (Median)	1.16	2.74	0.45	0.091 ^3^
Inpatient treatment (%)	48.0%	48.0%	81.6%	<0.001 ^1^
EDE score (Mean, SD)	3.27 (1.62)	3.32 (1.39)	3.22 (1.36)	0.945 ^2^
EDI-2 score (Mean, SD)	67.32 (39.55)	69.62 (38.79)	91.57 (47.35)	0.009 ^2^
FEICS–CC score (Mean, SD)	12.15 (4.17)	12.52 (4.44)	13.51 (4.08)	0.272 ^2^
FEICS–EOI score (Mean, SD)	23.92 (4.43)	23.53 (4.17)	22.91 (3.94)	0.503 ^2^

^1^ Chi² test; ^2^ ANOVA test; ^3^ Kruskal–Wallis test. Abbreviations: AN, anorexia nervosa; CC, criticism; COMP, comparison group; ED, eating disorder; EDE, Eating Disorder Examination Interview, EDI-2, Eating Disorder Inventory-2; EOI, emotional overinvolvement; FEICS, Family Emotional Involvement and Criticism Scale; FQ, Family Questionnaire; ONL, online; SUCCEAT, Supporting Carers of Children and Adolescents with Eating Disorders in Austria; WS, workshop.

**Table 2:** Multivariate regression (forward selection method) predicting criticism and emotional overinvolvement (FQ, parents).

Predictor	b(SE)	*t* (df)	*p*	*R*²	Δ*R*²
Outcome: FQ, criticism					
Model 1				0.351	
CASK, total score	−0.240 (0.029)	−8.165 (1,123)	<0.001		
Model 2				0.435	0.084
CASK, total score	−0.171 (0.032)	−5.378	<0.001		
EDSIS, total score	0.127 (0.030)	4.253 (2,122)	<0.001		
Model 3				0.462	0.27
CASK, total score	−0.160 (0.032)	−5.079	<0.001		
EDSIS, total score	0.137 (0.030)	4.624	<0.001		
Treatment type ^1^	1.849 (0.755)	2.450 (1,121)	0.016		
Outcome: FQ, emotional overinvolvement					
Model 1				0.500	
EDSIS, total score	0.253 (0.023)	11.035 (1,122)	<0.001		
Model 2				0.567	0.068
EDSIS, total score	0.195 (0.025)	7.741	<0.001		
BDI, total score	0.203 (0.047)	4.354 (2,121)	<0.001		
Model 3				0.601	0.033
EDSIS, total score	0.173 (0.025)	6.863	<0.001		
BDI, total score	0.195 (0.045)	4.320	<0.001		
URICA, contemp.	0.173 (0.055)	3.170 (3,120)	0.002		
Model 4				0.620	0.020
EDSIS, total score	0.154 (0.026)	5.956	<0.001		
BDI, total score	0.160 (0.046)	3.437	0.001		
URICA, contemp.	0.169 (0.054)	3.164	0.002		
CASK, total score	−0.064 (0.026)	−0.2486 (4,119)	0.014		
Model 5				0.635	0.015
EDSIS, total score	0.137 (0.027)	5.119	<0.001		
BDI, total score	0.101 (0.053)	1.916	0.058		
URICA, contemp.	0.162 (0.053)	3.074	0.003		
CASK, total score	−0.061 (0.025)	−2.398	0.018		
GHQ, total score	0.248 (0.113)	2.185 (5,118)	0.031		

^1^ 1 = inpatient, 2 = outpatient. Abbreviations: BDI, Beck Depression Inventory; CASK, Caregiver Skills Scale; contemp., contemplation; EDSIS, Eating Disorder Impact Scale; FQ, Family Questionnaire; GHQ, General Health Questionnaire; URICA, University of Rhode Island Change Assessment.

**Table 3:** Multivariate regression (forward selection method) predicting criticism and emotional overinvolvement (FEICS, patients).

Predictor	b(SE)	*t* (df)	*p*	*R*²	Δ*R*²
Outcome: FEICS, criticism					
Model 1 (final)				0.145	
EDI-2, total score	0.040 (0.009)	4.555 (1,122)	<0.001		
Outcome: FEICS, emotional overinvolvement					
Model 1				0.041	
BMI percentile ^1^	−1.681 (0.701)	−2.399 (1,136)	0.018		
Model 2				0.085	0.044
BMI percentile ^1^	−2.025 (0.701)	−2.894	0.004		
URICA, precon.	−0.191 (0.074)	−2.563 (2,135)	0.011		

^1^ Categorized as follows: 1 ≤ 1st percentile, 2 > 1st percentile. Abbreviations: EDI-2, Eating Disorder Inventory-2; FEICS, Family Emotional Involvement and Criticism Scale; precon., precontemplation; URICA, University of Rhode Island Change Assessment.

**Table 4:** Results of linear regression analyses predicting the change in patient’s eating disorder symptoms by the change in high expressed emotion.

	EDE Change Score		EDI-2 Change Score		BMI Perc. Change	
	T0–T1	T0–T2	T0–T1	T0–T2	T0–T1	T0–T2
Change in CC (FQ)	*p* = 0.731	*p* = 0.239	*p* = 0.040	*p* = 0.073	*p* = 0.995	*p* = 0.099
Change in EOI (FQ)	*p* = 0.515	*p* = 0.148	*p* = 0.113	*p* = 0.050	*p* = 0.969	*p* = 0.066
Change in CC (FEICS)	*p* = 0.040	*p* = 0.087	*p* = 0.005	*p* < 0.001	*p* = 0.006	*p* = 0.561
Change in EOI FEICS)	*p* = 0.096	*p* = 0.438	*p* = 0.580	*p* = 0.867	*p* = 0.982	*p* = 0.307

Abbreviations: CC, criticism, EOI, emotional overinvolvement, EDE, Eating Disorder Examination Interview, EDI-2, Eating Disorder Inventory-2, FQ, Family Questionnaire, FEICS, Family Emotional Involvement and Criticism Scale; T0–T1, difference between the baseline and post-intervention scores; T0-T2, difference between the baseline and 12-month FU scores.

## Supplementary Materials

The following are available online at https://www.mdpi.com/2077-0383/9/7/2021/s1: Supplementary Tables S1 and S2.
