# Supplementary material for: Reduction of High Expressed Emotion and Treatment Outcomes in Anorexia Nervosa—Caregivers’ and Adolescents’ Perspective
Source: J Clin Med. 2020 Jun 27;9(7):2021. doi: 10.3390/jcm9072021 (PMC7409203; doi:10.3390/jcm9072021)
Supplement: Supplementary file 1 [file jcm-09-02021-s001.pdf]

Journal of Clinical Medicine

MDPI

**Table S1.** Univariate linear regression predicting criticism and emotional overinvolvement (FQ parents) - slopes presented only.

| Predictor | FQ-CC b(SE) | *t*(*p*) | FQ-EOI b(SE) | *t*(*p*) |
|---|---|---|---|---|
| Female sex (parent) [1] | -1.921(.191) | -1.613 (.109) | -1.826 (.092) | -1.672 (.097) |
| Age parent | 0.043 (.099) | 0.433 (.666) | 0.058 (.090) | 0.640 (.523) |
| GHQ total score | 0.496 (.117) | 4.239 (**<.001**) | 0.852 (.621) | 9.541 (**<.001**) |
| EDSIS total score | 0.220 (.026) | 8.458 (**<.001**) | 0.250 (.021) | 12.171 (**<.001**) |
| CASK total score | -0.204(.029) | -7.145 (**<.001**) | -0.183 (.497) | -6.927 (**<.001**) |
| BDI total score | 0.202 (.059) | 3.414 (**.001**) | 0.393 (.032) | 8.568 (**<.001**) |
| STAI state score | 0.133 (.039) | 3.400 (**.001**) | 0.237 (.032) | 7.513 (**<.001**) |
| STAI trait score | 0.154 (.044) | 3.472 (**.001**) | 0.261 (.037) | 7.115 (**<.001**) |
| URICA precontemp. | -0.125(.099) | -1.253 (.212) | -0.270 (.089) | -3.036 (**.003**) |
| URICA contemp. | 0.213 (.078) | 2.721 (**.007**) | 0.340 (.067) | 5.057 (**<.001**) |
| URICA action | 0.242 (.087) | 2.775 (**.006**) | 0.274 (.078) | 3.498 (**.001**) |
| Time spent caregiving [2] | 0.538 (.995) | 0.540 (.590) | 0.739 (.940) | 0.786 (.433) |
| Age patient | 0.233 (.275) | 0.849 (.397) | 0.419 (.250) | 1.675 (.096) |
| Treatment type [3] | 1.993 (.914) | 2.180 (**.031**) | 0.852 (.850) | 1.003 (.317) |
| ED duration | 0.062 (.034) | 1.807 (.073) | 0.038 (.032) | 1.207 (.230) |
| BMI Perc.cat [4] | 1.220 (.910) | 1.341 (.182) | 0.852 (.833) | 1.022 (.308) |
| EDE total score | -0.386(.318) | -1.212 (.227) | 0.068 (.847) | 0.080 (.936) |
| EDI total score | 0.007 (.011) | 0.674 (.501) | 0.014 (.010) | 1.477 (.142) |

[1] 1= female, 2 = male; [2] 1 = < 3h/day, 2 = ≥ 3h/day; [3] 1 = inpatient, 2 = outpatient, [4] 1 = ≤ 1st percentile, 2 = > 1st percentile; Abbreviations: BDI Beck Depression Inventory, CASK Caregiver Skills Scale, contemp. contemplation, EDE Eating Disorder Examination Interview, EDI-2 Eating Disorder Inventory-2, EDSIS Eating Disorders Symptoms Impact Scale, FQ-CC Family Questionnaire Criticism Score, FQ-EOI Family Questionnaire Emotional Overinvolvement Score, GHQ General Health Questionnaire, Perc.cat Percentile category, STAI State/Trait Anxiety Inventory, URICA University of Rhode Island Change Assessment Scale.

**Table S2.** Univariate linear regression predicting criticism and emotional overinvolvement (FEICS, patients) - slopes presented only.

| **Predictor** | **FEICS-CC b(SE)** | $t(p)$ | **FEICS-EOI b(SE)** | $t(p)$ |
|---|---|---|---|---|
| Age parent | 0.102 (.074) | 1.381 (.170) | 0.120 (.073) | 1.646 (.102) |
| GHQ total score | 0.043 (.096) | 0.450 (.654) | 0.149 (.093) | 1.596 (.113) |
| EDSIS total score | 0.039 (.025) | 1.563 (.120) | 0.039 (.024) | 1.584 (.115) |
| CASK total score | -0.047 (.026) | -1.809 (.073) | -0.005 (.026) | -0.196 (.845) |
| BDI total score | 0.011 (.049) | 0.223 (.824) | 0.073 (.049) | 1.510 (.134) |
| STAI state score | -0.048 (.031) | -1.562 (.121) | 0.036 (.031) | 1.136 (.258) |
| STAI trait score | 0.030 (.035) | 0.859 (.392) | 0.030 (.036) | 0.852 (.396) |
| URICA precontemp. | 0.035 (.079) | 0.451 (.653) | -0.149 (.075) | -1.992 (**.048**) |
| URICA contemp. | -0.082 (.062) | -1.323 (.188) | 0.090 (.060) | 1.494 (.138) |
| URICA action | -0.052 (.070) | -0.750 (.454) | 0.106 (.067) | 1.588 (.115) |
| Time spent caregiving [1] | -0.640 (.799) | -0.802 (.424) | 1.359 (.774) | 1.755 (.081) |
| Age patient | 0.275 (.210) | 1.304 (.194) | 0.235 (.208) | 1.130 (.260) |
| Treatment type [2] | 0.731 (.721) | 1.014 (.312) | -0.739 (.710) | -1.041 (.299) |
| ED duration | 0.062 (.029) | 2.134 (**.035**) | -0.035 (.029) | -1.227 (.222) |
| BMI Perc.cat [3] | 0.432 (.709) | 0.609 (.544) | -1.574 (.686) | -2.293 (**.023**) |
| EDE total score | 0.777 (.243) | 3.202 (**.002**) | -0.240 (.246) | -0.975 (.331) |
| EDI total score | 0.039 (.008) | 5.098 (**<.001**) | -0.009 (.008) | -1.083 (.281) |

[1] 1 = < 3h/day, 2 = ≥ 3h/day; [2] 1 = inpatient, 2 = outpatient, [3] 1 = ≤ 1st percentile, 2 = > 1st percentile; Abbreviations: BDI Beck Depression Inventory, CASK Caregiver Skills Scale, contemp. contemplation, EDE Eating Disorder Examination Interview, EDI-2 Eating Disorder Inventory-2, EDSIS Eating Disorders Symptoms Impact Scale, FQ-CC Family Questionnaire Criticism Score, FQ-EOI Family Questionnaire Emotional Overinvolvement Score, GHQ General Health Questionnaire, Perc.cat Percentile category, precontemp. precontemplation, STAI State/Trait Anxiety Inventory, URICA University of Rhode Island Change Assessment Scale.
